# Psychometric evaluation of the Turkish version of the Beverage Intake Questionnaire-15

**DOI:** 10.3389/fnut.2025.1721223

**Published:** 2026-01-05

**Authors:** Cansu Gencalp, Olcay Baris, Ece Ones, Simge Sipahi, Meryem Kahriman, Nihan Cakir Bicer, Salim Yilmaz, Murat Bas

**Affiliations:** 1 Department of Nutrition and Dietetics, Faculty of Health Sciences, Acibadem Mehmet Ali Aydinlar University, Istanbul, Türkiye; 2 Department of Nutrition and Dietetics, Institute of Health Sciences, Acibadem Mehmet Ali Aydinlar University, Istanbul, Türkiye; 3 Department of Molecular Gastroenterology and Hepatology, Institute of Gastroenterology and Hepatology, Kocaeli University, Kocaeli, Türkiye; 4 Department of Healthcare Management, Faculty of Health Sciences, Acibadem Mehmet Ali Aydinlar University, Istanbul, Türkiye

**Keywords:** beverages, FFQ, food frequency questionnaire, nutrition assessment, sugar-sweetened beverages

## Abstract

**Background/objectives:**

Overweight and obesity remain major unresolved public health issues. One contributing factor to the increasing prevalence of obesity is the rising consumption of sugar-sweetened beverages (SSBs), which are a key source of added sugars and excess energy in the diet. Therefore, accurate assessment of beverage intake has become increasingly important for both research and public health interventions. This study aimed to evaluate the validity and reliability of the Turkish version of the Beverage Intake Questionnaire-15 (BEVQ-15) in adults.

**Methods:**

A total of 141 adults aged 18–65 years who applied to a private nutrition clinic were included. Data were collected at three time points (T1, T2, T3) through face-to-face interviews. At each time point, participants completed the BEVQ-15 and a 3-day food record. Validity and reliability were evaluated using Spearman correlations, intraclass correlation coefficients, Kendall’s W, and Bland–Altman analyses.

**Results:**

Spearman correlations between BEVQ-15 and food records ranged from 0.26 to 0.96 across beverage categories, with the majority of categories demonstrating correlations of 0.50 or higher. Test–retest reliability was excellent for water (ICC = 0.94), moderate for diet beverages (ICC = 0.70), and total SSBs (ICC = 0.65). Bland–Altman analysis showed that 87% of beverage categories achieved ≥95% agreement within the limits of agreement.

**Conclusion:**

These findings indicate that the Turkish version of the BEVQ-15 is a valid and reliable instrument for assessing beverage consumption among adults.

## 1 Introduction

Overweight and obesity are among the most significant unresolved public health issues, with their global incidence roughly quadrupling over the past four decades (1). According to the Global Obesity Observatory, Türkiye’s adult obesity prevalence is 34.4%, placing the country 39th in the global rankings (2).

Increased consumption of sugar-sweetened beverages (SSBs) has coincided with the rising prevalence of obesity (3). Although an exact definition is lacking, drinks that contain free sugar or added sugar are generally regarded as SSBs. Soft drinks, fruit juices, energy drinks, and some caffeinated beverages fall under this classification (4). According to the Centers for Disease Control and Prevention (CDC, 2024), drinks including sodas, fruit drinks, sports, and energy drinks account for about 24% of total added sugar intake in the American diet, making SSBs the single largest source of added sugars (5). SSBs lead to weight gain by increasing the amount of liquid energy consumed, causing hyperinsulinemia due to the rapid absorption of glucose and possibly stimulating the dopaminergic reward system (1). Due to these effects, SSBs are positioned as an important risk factor for noncommunicable diseases such as type 2 diabetes mellitus, ischemic heart disease, and metabolic syndrome (6).

The World Health Organization (7) recommended that free sugar intake should be less than 10% of total daily energy intake, with a conditional suggestion that reducing it to below 5% may provide additional health benefits. The American Heart Association recommends limiting added sugars to no more than 6% of total daily energy intake, which corresponds to approximately 100 kcal per day (about 6 teaspoons) for women and 150 kcal per day (about 9 teaspoons) for men. The AHA emphasizes that these recommendations apply to all sources of added sugars and do not single out specific types such as high-fructose corn syrup; however, sugar-sweetened beverages remain the largest contributor, accounting for nearly half of added sugars in the American diet (8). Yan et al. specifically debated whether the WHO recommendation should be reduced from 10 to 5%, highlighting the ongoing controversy surrounding the optimal threshold for free sugar intake (9). Although a decline in the consumption of SSBs has been observed since 2000, they remain one of the leading sources of free sugars in the Western diet (1, 10).

According to a modeling study published in 2025, worldwide, SSB consumption was estimated to contribute to 2.2 million new type 2 diabetes cases and 1.2 million cardiovascular disease events in 2020, representing 9.8 and 3.1% of the total global incidence of these diseases, respectively (11). The study also reported that low- and middle-income countries were affected more severely because SSB consumption rates are increasing rapidly in these regions (11). The US Dietary Guidelines have addressed this increasing health burden by recommending that saturated fats and added sugars should not exceed 15% of an individual’s daily energy intake and suggesting water or low-fat milk as healthier alternatives to SSBs (12). Evidence from a 2023 systematic review and meta-analysis that included 51 prospective cohort studies and 34 randomized controlled trials indicated that SSB consumption is positively associated with weight gain and increased body mass index (BMI) in children and adults (13).

Given the substantial evidence linking SSB intake to adverse health outcomes, accurate methods are required for the assessment of beverage consumption patterns. The detailed intake 3-day food record comes with the drawbacks of being time-consuming and resource-intensive while failing to show actual habitual consumption patterns (14). In contrast, food frequency questionnaires, such as the BEVQ-15, provide a practical and validated method for assessing long-term beverage consumption patterns (14, 15). Fausnacht et al. and Hedrick et al. (15, 16) evaluated the validity and reliability of the Turkish version of the BEVQ-15 for use in adult populations. Although the BEVQ-15 has been validated in several languages, no short and validated tool currently exists in Turkish to assess habitual beverage intake. Considering the rising prevalence of overweight and obesity in Türkiye and the growing contribution of SSBs to the national diet, adapting the BEVQ-15 into Turkish is essential to provide researchers and practitioners with a culturally appropriate, practical, and reliable assessment instrument.

## 2 Materials and methods

### 2.1 Participants and study design

This observational study was conducted between May and July 2025 among 141 adult individuals (≥18 years) who applied to a private nutrition education and consultancy clinic. The exclusion criteria included individuals under the age of 18 or over the age of 65, pregnant or breastfeeding women, those with chronic diseases, and individuals following a specific diet program for any reason.

The study was carried out in accordance with the ethical principles of the Declaration of Helsinki. Ethical approval was obtained from the Acibadem Mehmet Ali Aydinlar University Medical Research Ethics Committee (ATADEK-2025-07/314). Participation was entirely voluntary, and written informed consent was obtained from all participants.

### 2.2 Data collection

Data were collected from participants via face-to-face interviews conducted at three different time points (T1, T2, and T3), each separated by 15-day intervals. Demographic characteristics (age, gender, education level, and employment status) of participants were recorded at the beginning of the study (T1), and body weight and height were measured at both the beginning (T1) and the end (T3). Body mass index (BMI) was calculated using the formula body weight (kg)/height (m^2^) at T1 and T3.

At all three time points (T1, T2, and T3), participants were asked to complete the BEVQ-15 questionnaire. In addition, 3-day dietary records were collected from each participant at every time point to assess the correlation between the questionnaire results and actual beverage consumption.

#### 2.2.1 Beverage intake questionnaire (BEVQ)-15

The BEVQ is a 19-item quantitative food consumption frequency form designed in 2010 to assess the frequency and amount of beverages consumed by an individual (14). This scale was initially planned to have 19 items, but analysis led to the decision to eliminate the vegetable juice, meal replacement drinks, and mixed alcoholic drinks categories and combine the beer and light beer categories into a single category. In this direction, a 15-item version was developed in 2012. It was also found that this structure allows for rapid estimation of beverage consumption (15). In 2020, Fausnacht et al. (16) updated the form to include only 15 items including the following beverage categories: water or unsweetened sparkling water, 100% fruit juice, sweetened juice beverage/drink, whole milk, reduced fat milk (2%) or chocolate milk, low fat milk (1%), fat free/skim milk, buttermilk or soy milk, nut milk (almond, cashew, coconut) regular soft drinks, regular energy and sports drinks, diet or artificially sweetened soft drinks and energy/sports drinks, sweet tea (with sugar), black tea or coffee (no creamer or milk), tea or coffee with milk and/or creamer, wine (red or white), hard liquor, beer, ales, wine coolers, non-alcoholic or light beer, and other beverages.

The BEVQ-15 assesses an individual’s frequency and quantity of water, sugary drinks, other beverages, and total beverage intake in grams (g) and kilocalories (kcal). The frequency (“How often?”) is converted to units per day, then multiplied by the amount consumed each time (“How much each time?”) to obtain the average daily beverage consumption (in mL).

In addition to total beverage consumption, to quantify total SSB consumption, beverage categories containing added sugars were summed (sweetened juice beverage/drinks, regular soft drinks, regular energy and sports drinks, tea sweetened with sugar, and tea and/or coffee sweetened with sugar and/or sweetened creamer).

The version used in this study was based on a 15-item structure, but was revised by adding items such as ayran and kefir, considering the beverage consumption habits of the Turkish society.

#### 2.2.2 3-day food record

Three lots of 3-day food records were taken from each participant, one at each of the three time points of the study. For each individual, two of the 3-day records were taken on weekdays, and one was taken over a weekend. Before the food records were administered, a face-to-face training interview was conducted with each individual, and detailed information was provided on portion control, consumption frequency, cooking methods, and points to be considered during the recording. Participants were asked to note all food and drink they consumed throughout the day, and examples were given using household measurements (glass, tablespoon, slice, etc.) for the standardization of measurement units. The obtained data were analyzed using Nutrition Information System software (BeBiS 7.2, Ebispro for Windows, Stuttgart, Germany). The participants’ daily total energy and the amount of energy they consumed via beverage intake were calculated using the same software.

### 2.3 Statistical analysis

The sample size was determined using G*Power 3.1.9.7 with parameters set for correlation analysis at an expected medium effect size (*r* = 0.50 from previous BEVQ validation studies), *α* = 0.05, power = 0.80, and two-tailed testing, yielding a minimum of 84 participants; to accommodate potential dropouts, 141 adults were recruited, which provided observed power ≥0.99 for detecting correlations ≥0.35. Statistical analyses were performed in R 4.4.2 (R Foundation for Statistical Computing, Vienna, Austria) using the pwr, irr, boot, cocor, ggplot2, and BlandAltmanLeh packages. Normality was assessed using Shapiro–Wilk tests for each beverage category. Given that 87% of variables showed non-normal distributions (*p* < 0.05), non-parametric statistical methods were employed throughout the analysis. Intraclass correlation coefficients (ICC) were interpreted using commonly accepted thresholds, where values below 0.50 indicate poor reliability, values between 0.50 and 0.75 indicate moderate reliability, values between 0.75 and 0.90 indicate good reliability, and values above 0.90 indicate excellent reliability. Convergent validity was assessed using Spearman correlations between BEVQ-15 and 3-day food records at each time point, while test–retest reliability was evaluated through ICC (1, 2) absolute agreement models, bootstrap ICCs with 1,000 replications for robust confidence intervals, and Kendall’s W for ranking consistency. Agreement between methods was further examined with Bland–Altman analyses, where proportional bias was tested by regressing differences on means (*p* < 0.05 = significant bias), and subgroup analyses compared sugar-sweetened beverages (regular soft drinks, sweetened fruit drinks, sweet tea, energy/sports drinks, sweetened coffee/tea) with non-SSBs using Mann–Whitney U tests. All tests were two-tailed with *α* = 0.05, and no correction for multiple comparisons was applied, as each beverage category represented an independent assessment.

## 3 Results

The study included 141 participants who completed both the food records and the BEVQ-15 questionnaire at three distinct time points. Among the 141 individuals who participated in the study, 64.54% were female and 35.46% were male. Regarding educational status, 73.05% were university graduates, 13.48% held a master’s or doctoral degree, and 13.48% had a high school education or lower. In terms of occupation status, 53.90% were employed, 39.72% were students, 5.67% were unemployed, and 0.71% were retired. With respect to marital status, 73.76% of the participants were unmarried and 26.24% were married. Based on the BMI classification, 13.48% of participants were underweight, 47.52% had normal weight, 26.24% were overweight, and 12.77% were obese. Regarding income level, 54.61% reported their income as equal to their expenses, 34.75% indicated their income exceeded their expenses, and 10.64% stated their income was less than their expenses. The participants’ ages ranged from 19 to 63 years, with a mean age of 30.65 ± 10.16 years. The BMI values ranged from 16.16 to 61.35 kg/m^2^, with a mean BMI of 24.16 ± 5.91 kg/m^2^. Table 1 shows the demographic data and anthropometric measurements of the participants included in this study.

**Table 1:** Demographic data and anthropometric measurements of the participants.

Demographic characteristics	x̄ ± SS	Min-Max
Age	30.65 ± 10.16	19–63
BMI	24.16 ± 5.91	16.16–61.35
	*N*	%
Gender		
Male	50	35.46
Female	91	64.54
Education		
High school or below	19	13.48
Undergraduate (Bachelor’s degree)	103	73.05
Graduate degree (Master’s/PhD)	19	13.48
Occupation		
Employed	76	53.9
Student	56	39.72
Unemployed	8	5.67
Retired	1	0.71
Marital status		
Married	37	26.24
Single	104	73.76
Income		
Income is less than expenses	15	10.64
Income is equal to expenses	77	54.61
Income is higher than expenses	49	34.75
BMI classification		
Underweight	19	13.48
Normal weight	67	47.52
Overweight	37	26.24
Obesity	18	12.77
Total	141	100

Table 2 shows the convergent validity between the BEVQ-15 and the 3-day food record across the three time points for various beverage categories. The Spearman’s Rho (*ρ*) coefficients ranged from 0.260 to 0.963, indicating varying levels of agreement across beverage types. Water intake (measured in mL) exhibited consistently high correlations (*ρ* = 0.850–0.919) across all assessments. Nut milk consumption (measured in mL and kcal) and sweet tea intake (mL and kcal) also demonstrated high correlations, with *ρ* values ranging from 0.793 to 0.963. Meanwhile, energy and sports drinks (mL and kcal) showed ρ values between 0.678 and 0.784. Regular soft drinks, sweetened fruit drinks, and milk-based beverages (whole milk and low-fat/fat-free milk) presented moderate correlations, with *ρ* values between 0.551 and 0.692. In contrast, coffee/tea sweetened/milk/cream, coffee/tea black, and beer exhibited lower correlations (*ρ* = 0.260–0.552). Total SSBs intake displayed ρ values ranging from 0.368 to 0.400 for volume and 0.469 to 0.545 for energy. The total beverage intake correlations were moderate, with ρ values between 0.358 and 0.451 for volume and 0.510 to 0.580 for energy.

**Table 2:** Convergent validity based on the BEVQ-15 questionnaire and the 3-day food record.

Beverage category	BEVQ-15-1 Mean (SD)	Food Record 1 Mean (SD)	Difference	rho (ρ) coefficient	BEVQ-15-2 Mean (SD)	Food Record 2 Mean (SD)	Differences	rho (ρ) coefficient	BEVQ-15-3 Mean (SD)	Food Record 3 Mean (SD)	Differences	rho (ρ) coefficient
Water (mL)	610.50 (216.59)	645.69 (285.10)	−35.191	0.850	550.35 (232.36)	585.55 (296.46)	−35.199	0.905	553.90 (233.47)	587.32 (298.25)	−33.418	0.919
100% Fruit Juice (mL)	11.21 (27.92)	4.23 (15.80)	6.986	0.583	11.89 (46.46)	5.82 (44.48)	6.064	0.455	8.88 (24.55)	3.63 (19.67)	5.248	0.555
100% Fruit Juice (kcal)	6.62 (16.48)	3.06 (10.42)	3.551	0.520	7.01 (27.41)	3.44 (26.25)	3.578	0.455	5.24 (14.49)	2.41 (11.99)	2.827	0.589
Sweetened fruit drink (mL)	19.15 (58.43)	5.30 (19.86)	13.854	0.620	11.90 (28.91)	4.01 (13.75)	7.889	0.625	12.84 (32.35)	3.23 (11.68)	9.607	0.612
Sweetened fruit drink (kcal)	9.19 (28.05)	5.30 (19.86)	3.896	0.620	5.71 (13.88)	4.01 (13.75)	1.700	0.625	6.16 (15.53)	3.23 (11.68)	2.931	0.612
Whole milk, reduced fat milk, or chocolate milk (mL)	24.28 (53.31)	16.58 (45.66)	7.702	0.628	27.89 (57.10)	17.02 (42.52)	10.870	0.651	26.21 (55.93)	17.63 (46.40)	8.580	0.690
Whole milk, reduced fat milk, or chocolate milk (kcal)	16.03 (35.18)	10.95 (30.15)	5.076	0.628	18.41 (37.68)	11.24 (28.08)	7.167	0.651	17.30 (36.92)	11.95 (31.17)	5.347	0.692
Low-fat or fat-free milk, buttermilk, or soy milk (mL)	21.13 (63.29)	6.18 (21.09)	14.950	0.551	21.60 (67.55)	7.18 (21.90)	14.411	0.637	19.40 (55.55)	8.59 (28.56)	10.809	0.651
Low-fat or fat-free milk, buttermilk, or soy milk (kcal)	8.45 (25.32)	3.48 (10.45)	4.974	0.549	8.64 (27.02)	4.11 (11.52)	4.531	0.679	7.76 (22.22)	4.51 (12.53)	3.244	0.662
Nut milk (mL)	11.40 (51.42)	10.58 (51.06)	0.823	0.864	9.17 (49.58)	12.13 (65.61)	−2.957	0.837	10.50 (50.52)	5.65 (22.78)	4.858	0.794
Nut milk (kcal)	3.61 (16.87)	3.38 (16.79)	0.228	0.863	2.88 (16.06)	3.89 (21.46)	−1.019	0.835	3.28 (16.36)	1.74 (7.24)	1.539	0.793
Regular soft drink (mL)	40.79 (80.05)	27.84 (73.92)	12.957	0.670	37.38 (68.67)	23.52 (55.42)	13.851	0.624	40.80 (81.22)	26.50 (73.32)	14.305	0.652
Regular soft drink (kcal)	17.95 (35.22)	15.06 (33.41)	2.885	0.668	16.45 (30.21)	14.91 (37.70)	1.533	0.668	17.95 (35.74)	14.48 (41.60)	3.471	0.647
Energy and sports drinks (mL)	7.33 (22.48)	5.35 (20.99)	1.972	0.784	8.05 (31.21)	6.00 (31.58)	2.048	0.678	5.64 (18.13)	4.28 (18.63)	1.362	0.729
Energy and sports drinks (kcal)	3.44 (10.56)	2.52 (9.87)	0.927	0.784	3.78 (14.67)	2.81 (14.79)	0.976	0.678	2.65 (8.52)	2.00 (8.69)	0.652	0.729
Diet or artificially flavored sweetened drinks (mL)	39.91 (197.33)	26.63 (153.61)	13.284	0.796	21.67 (100.40)	15.84 (96.29)	5.830	0.734	43.21 (202.53)	29.48 (160.34)	13.723	0.792
Diet or artificially flavored sweetened drinks (kcal)	0.40 (1.97)	0.27 (1.54)	0.133	0.796	0.22 (1.00)	0.16 (0.96)	0.058	0.734	0.43 (2.03)	0.29 (1.60)	0.137	0.792
Sweet tea (mL)	18.21 (58.20)	13.44 (37.74)	4.770	0.952	10.48 (26.49)	7.77 (20.64)	2.716	0.882	12.41 (32.12)	11.33 (30.27)	1.078	0.956
Sweet tea (kcal)	36.26 (157.46)	25.78 (88.84)	10.479	0.963	18.43 (66.24)	10.66 (41.33)	7.766	0.875	16.94 (65.34)	16.38 (65.32)	0.553	0.952
Coffee/tea black (mL)	323.73 (485.34)	209.99 (372.04)	113.738	0.482	259.76 (409.22)	170.29 (289.16)	89.467	0.447	290.38 (404.26)	193.43 (347.66)	96.950	0.508
Coffee/tea black (kcal)	0.42 (1.07)	0.75 (1.49)	−0.329	0.519	0.42 (1.07)	0.73 (1.60)	−0.310	0.511	0.51 (1.14)	0.62 (1.37)	−0.113	0.483
Coffee/tea sweetened/milk/crea m (mL)	170.16 (263.95)	64.64 (158.63)	105.526	0.437	54.55 (122.50)	14.33 (67.43)	40.216	0.260	72.63 (218.58)	13.69 (63.15)	58.943	0.442
Coffee/tea sweetened/milk/cream (kcal)	50.91 (75.83)	21.78 (52.27)	29.129	0.436	13.53 (32.65)	5.69 (23.71)	7.832	0.269	20.07 (56.46)	8.42 (36.83)	11.647	0.405
Wine (mL)	4.91 (15.68)	4.47 (15.53)	0.447	0.792	5.63 (14.58)	4.54 (14.63)	1.095	0.680	14.58 (65.82)	9.98 (48.74)	4.606	0.707
Wine (kcal)	3.39 (10.82)	4.52 (18.00)	−1.126	0.787	3.89 (10.06)	4.53 (18.14)	−0.642	0.673	10.06 (45.41)	8.67 (38.20)	1.388	0.698
Hard liquor (mL)	5.70 (15.70)	6.62 (20.56)	−0.921	0.592	35.28 (266.86)	18.76 (189.86)	16.516	0.381	15.99 (71.34)	11.43 (56.85)	4.551	0.689
Hard liquor (kcal)	12.94 (35.64)	10.07 (38.50)	2.877	0.611	80.08 (605.78)	39.73 (430.22)	40.349	0.391	36.29 (161.95)	23.77 (128.42)	12.520	0.699
Beer (mL)	24.01 (55.29)	21.07 (50.24)	2.943	0.468	17.39 (44.55)	16.35 (44.48)	1.043	0.327	31.33 (77.48)	26.76 (68.52)	4.574	0.509
Beer (kcal)	8.16 (18.80)	8.65 (21.19)	−0.483	0.498	5.91 (15.15)	7.95 (22.85)	−2.032	0.343	10.65 (26.34)	10.70 (25.88)	−0.047	0.552
Total sugar-sweetened beverages (mL)	270.99 (307.71)	506.40 (828.35)	−235.404	0.392	154.01 (228.88)	395.33 (662.87)	−241.328	0.400	158.33 (331.68)	411.66 (839.96)	−253.333	0.368
Total sugar-sweetened beverages (kcal)	80.94 (93.47)	90.89 (126.10)	−9.955	0.469	52.53 (77.30)	108.00 (455.47)	−55.474	0.545	52.96 (95.23)	81.86 (206.98)	−28.905	0.521
Total beverage intake (mL)	1138.40 (917.74)	379.28 (485.21)	759.119	0.451	845.55 (788.22)	292.75 (410.19)	552.807	0.358	900.65 (894.33)	289.14 (482.15)	611.504	0.363
Total beverage intake (kcal)	179.49 (140.07)	93.24 (113.23)	86.254	0.580	201.87 (629.47)	77.62 (100.22)	124.249	0.545	171.57 (268.99)	85.87 (161.96)	85.695	0.510

*ρ*, Spearman’s rho coefficient.

Table 3 presents the test–retest reliability and reproducibility of beverage intake estimates across three time points, based on ICCs, bootstrap ICC confidence intervals, and Kendall’s W coefficients. Water intake (mL) demonstrated excellent test–retest reliability (ICC = 0.942; 95% CI: 0.924–0.956; *p* < 0.001), corroborated by bootstrap confidence intervals (0.863–0.998). However, the non-significant Kendall’s W (0.083, *p* = 1.000) indicates poor ranking consistency, suggesting that while the instrument captures water intake accurately at each time point, individuals’ relative consumption varies day-to-day. Diet beverages showed moderate reliability (ICC = 0.703) with non-significant ranking consistency (W = 0.176, *p* = 1.000), while 100% fruit juice and whole milk categories exhibited moderate reliability (ICC range: 0.58–0.65) with variable ranking patterns. Regular soft drinks demonstrated poor ICC (0.436) but significant ranking consistency for volume (W = 0.422, *p* = 0.019), suggesting stable relative consumption patterns despite absolute measurement variability. Several categories showed poor reliability (ICC < 0.50), including nut milk (ICC = 0.163), sweet tea (ICC = 0.150), and coffee/tea sweetened with milk/cream (ICC = 0.143). The negligible ICCs combined with non-significant Kendall’s W values for these categories indicate both poor absolute agreement and unstable ranking patterns, likely reflecting variable day-to-day consumption. Notably, total SSB intake demonstrated moderate reliability (ICC = 0.654 for mL, 0.394 for kcal) but exceptional ranking consistency (W = 0.862, *p* < 0.001 for mL; W = 0.819, *p* < 0.001 for kcal), indicating that while absolute intake estimates varied, individuals consistently maintained their position relative to others—a critical finding for epidemiological applications where ranking is more important than precise quantification. Similarly, total beverage intake showed moderate ICC (0.520 for mL) but strong ranking consistency (W = 0.728, *p* < 0.001), supporting the instrument’s utility for classifying individuals by consumption levels. The divergence between ICC and Kendall’s W values across categories highlights an important distinction: ICC measures absolute agreement while Kendall’s W assesses ranking consistency. Categories with high ICC but low W (e.g., water) show good measurement precision but variable individual patterns, while those with moderate ICC but high W (e.g., total SSBs) maintain consistent population rankings despite measurement variability—the latter being more relevant for epidemiological studies focused on identifying high consumers.

**Table 3:** Test–retest reliability and consistency of beverage intake estimates across repeated measurements.

Beverage category	ICC	Lower CI (%95)	Upper CI (%95)	*p*-value	ICC (Bootstrap)	Boot lower CI (%95)	Boot upper CI (%95)	Kendall’s W	*p*-value
Water (mL)	0.942	0.924	0.956	3.96 × 10^−143^	0.942	0.863	0.998	0.083	1.000
100% Fruit Juice (mL)	0.653	0.572	0.725	3.80 × 10^−41^	0.643	0.461	0.772	0.311	0.704
100% Fruit Juice (kcal)	0.584	0.494	0.666	5.95 × 10^−32^	0.567	0.369	0.685	0.298	0.813
Sweetened fruit drink (mL)	0.336	0.231	0.442	3.14 × 10^−11^	0.406	0.109	0.713	0.432	0.010
Sweetened fruit drink (kcal)	0.319	0.214	0.427	2.48 × 10^−10^	0.370	0.124	0.676	0.299	0.801
Whole milk. Reduced fat milk. or chocolate milk (mL)	0.617	0.532	0.695	3.50 × 10^−36^	0.599	0.322	0.802	0.347	0.354
Whole milk. Reduced fat milk. or chocolate milk (kcal)	0.588	0.499	0.670	1.71 × 10^−32^	0.573	0.288	0.763	0.347	0.355
Low-fat or fat-free milk. Buttermilk. or soy milk (mL)	0.576	0.486	0.660	4.16 × 10^−31^	0.547	0.045	0.839	0.329	0.531
Low-fat or fat-free milk. Buttermilk. or soy milk (kcal)	0.441	0.340	0.540	3.01 × 10^−18^	0.449	0.014	0.744	0.315	0.663
Nut milk (mL)	0.163	0.061	0.273	6.46 × 10^−4^	0.161	−0.003	0.407	0.055	1.000
Nut milk (kcal)	0.146	0.045	0.256	1.87 × 10^−3^	0.146	0.010	0.344	0.055	1.000
Regular soft drink (mL)	0.436	0.334	0.535	7.99 × 10^−18^	0.440	0.137	0.684	0.422	0.019
Regular soft drink (kcal)	0.598	0.511	0.679	9.21 × 10^−34^	0.581	0.392	0.703	0.340	0.416
Energy and sports drinks (mL)	0.369	0.265	0.473	3.39 × 10^−13^	0.372	−0.010	0.797	0.161	1.000
Energy and sports drinks (kcal)	0.383	0.279	0.487	4.21 × 10^−14^	0.385	−0.053	0.795	0.161	1.000
Diet or artificially flavored sweetened drinks (mL)	0.703	0.630	0.767	2.39 × 10^−49^	0.719	0.506	0.949	0.176	1.000
Diet or artificially flavored sweetened drinks (kcal)	0.703	0.630	0.767	2.39 × 10^−49^	0.724	0.514	0.946	0.176	1.000
Sweet tea (mL)	0.150	0.049	0.260	1.43 × 10^−3^	0.246	−0.004	0.918	0.046	1.000
Sweet tea (kcal)	0.007	−0.083	0.110	4.37 × 10^−1^	0.167	−0.005	0.345	0.046	1.000
Coffee/tea black (mL)	0.547	0.453	0.634	8.34 × 10^−28^	0.538	0.375	0.650	0.581	1.27 × 10^−7^
Coffee/tea black (kcal)	0.575	0.485	0.659	5.72 × 10^−31^	0.561	0.366	0.745	0.361	0.238
Coffee/tea sweetened/milk/cream (mL)	0.143	0.043	0.253	2.24 × 10^−3^	0.157	0.028	0.309	0.356	0.274
Coffee/tea sweetened/milk/cream (kcal)	0.120	0.021	0.229	8.40 × 10^−3^	0.126	0.027	0.244	0.388	0.091
Wine (mL)	0.275	0.170	0.384	4.14 × 10^−8^	0.295	0.151	0.551	0.140	1.000
Wine (kcal)	0.352	0.247	0.457	3.76 × 10^−12^	0.356	0.191	0.546	0.154	1.000
Hard liquor (mL)	0.187	0.085	0.298	1.11 × 10^−4^	0.210	0.084	0.434	0.211	1.000
Hard liquor (kcal)	0.186	0.083	0.296	1.27 × 10^−4^	0.288	0.182	0.663	0.252	0.985
Beer (mL)	0.691	0.617	0.757	2.34 × 10^−47^	0.678	0.460	0.828	0.458	0.002
Beer (kcal)	0.385	0.281	0.488	3.32 × 10^−14^	0.380	0.131	0.622	0.416	0.025
Total sugar-sweetened beverages (mL)	0.654	0.574	0.726	2.25 × 10^−41^	0.650	0.549	0.740	0.862	1.34 × 10^−21^
Total sugar-sweetened beverages (kcal)	0.394	0.290	0.497	8.07 × 10^−15^	0.451	0.381	0.649	0.819	3.57 × 10^−19^
Total beverage intake (mL)	0.520	0.424	0.611	4.25 × 10^−25^	0.520	0.413	0.616	0.728	2.20 × 10^−14^
Total beverage intake (kcal)	0.349	0.244	0.455	5.28 × 10^−12^	0.379	0.321	0.624	0.738	6.89 × 10^−15^

**Figure 1:**
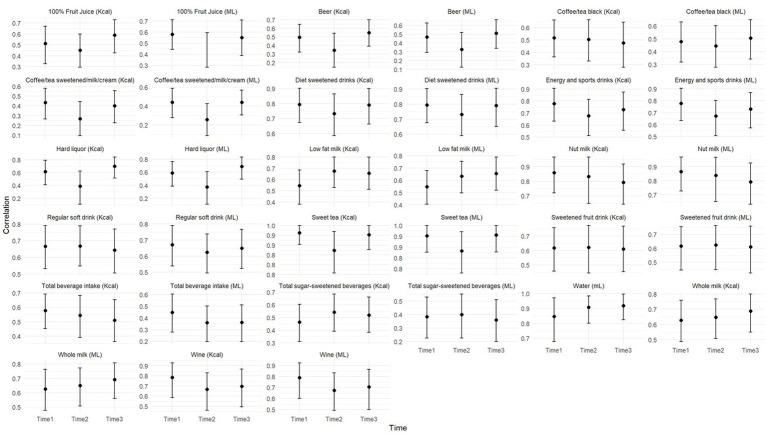
BEVQ-recall correlations and 95% confidence intervals over time.

Figure 1 illustrates the Spearman’s correlation coefficients between the BEVQ-15 and the 3-day food record beverage intake estimates across the three time points (T1, T2, T3) for all beverage categories, and the corresponding 95% bootstrap confidence intervals. Water intake (mL) exhibited consistently high correlations across all three time points (*ρ* = 0.85–0.92), supporting its excellent reproducibility with the highest ICC (0.942). This strong performance aligned with narrow confidence intervals shown in Figure 1. Sweet tea (mL) demonstrated the highest correlations among all categories (*ρ* = 0.88–0.96), though paradoxically showed poor test–retest reliability (ICC = 0.150), suggesting that while the instrument captures sweet tea consumption accurately at each time point, individual consumption patterns are highly variable. Nut milk showed strong correlations (*ρ* = 0.79–0.86) despite very low ICC (0.163), indicating good cross-sectional validity but inconsistent individual patterns over time. Diet sweetened drinks maintained high correlations (*ρ* = 0.73–0.80) with modarate reliability (ICC = 0.703), demonstrating both validity and temporal stability. Energy and sports drinks showed moderate to high correlations (*ρ* = 0.68–0.78) but lower reliability (ICC = 0.369–0.383). Regular soft drinks and sweetened fruit drinks demonstrated moderate correlations (*ρ* = 0.62–0.67 and 0.55–0.63 respectively), with corresponding poor to moderate ICC values (0.336–0.598). Notably, coffee/tea sweetened with milk/cream showed the poorest correlations (*ρ* = 0.26–0.44) and very low ICC (0.120–0.143), suggesting challenges in capturing these frequently consumed, variable-portion beverages. Total SSB intake showed surprisingly low correlations (*ρ* = 0.37–0.40) but moderate reliability (ICC = 0.654), while total beverage intake demonstrated similar patterns (*ρ* = 0.36–0.45, ICC = 0.520). The divergence between correlation coefficients and ICC values across categories highlights the distinction between cross-sectional validity and longitudinal reliability (Figure 1).

**Figure 2:**
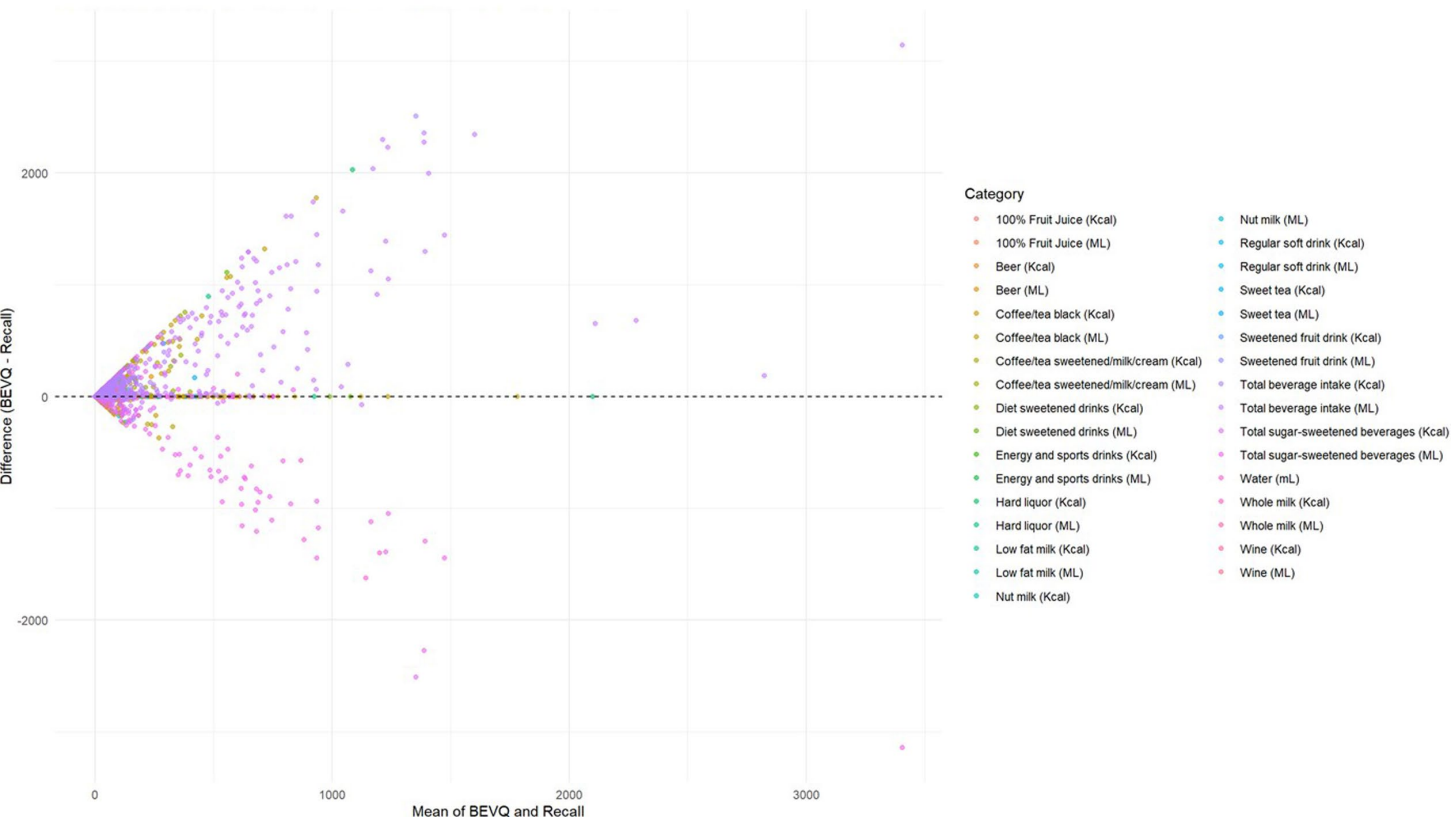
Bland–Altman plot for all beverage categories (average of 3 time points).

Figure 2 illustrates the Bland–Altman plot comparing BEVQ-15 and 3-day food record estimates across all beverage categories, averaged over three time points. The plot reveals a characteristic pattern with most data points clustering near the zero-difference line, particularly for lower consumption volumes. However, a notable funnel-shaped distribution is evident, with increasing scatter at higher mean intake levels. This pattern is particularly pronounced for high-volume categories such as water, coffee/tea, and total beverage intake, where differences exceed ±1,000 mL at the upper consumption ranges. The majority of observations fell within clinically acceptable ranges, though several outliers are visible, particularly in the total beverage and total SSB categories. The presence of both positive and negative differences across the consumption spectrum indicates no systematic directional bias, though the proportional bias (wider spread at higher intakes) is consistent with known challenges in dietary self-reporting. Categories with lower consumption volumes (diet drinks, alcoholic beverages, energy drinks) showed tighter clustering around the zero line, suggesting better agreement for infrequently consumed beverages. Despite the observed variability at higher intake levels, the overall pattern supports acceptable concordance between methods for population-level assessments, though individual-level precision may be limited for high consumers (Figure 2).

Bland–Altman analysis (Table 4) was performed to evaluate agreement between BEVQ-15 and 3-day food records, with acceptable agreement defined *a priori* as ≥95% of observations falling within limits of agreement (LoA = mean difference ± 1.96 SD). The analysis revealed generally good agreement across most beverage categories, with 87% (26/30) of categories meeting the acceptability criterion. Water intake demonstrated excellent agreement with minimal systematic bias (−34.6 mL) and 97.2% of observations within LoA. However, proportional bias was significant (*p* < 0.001), indicating greater variability at higher intake levels. This pattern of proportional bias was observed in 67% (20/30) of categories (*p* < 0.05), consistent with known challenges in dietary self-reporting where measurement error increases with consumption volume. Sugar-sweetened beverages (SSBs) versus non-SSB categories showed differential performance. Among SSB categories, mean agreement was 95.1% (range: 93.6–97.2%), with sweetened tea showing the smallest bias (2.9 mL, 97.2% within LoA) despite significant proportional bias. Regular soft drinks and energy/sports drinks showed moderate positive bias (13.7 mL and 1.8 mL respectively), both achieving 93.6% agreement. In contrast, non-SSB categories demonstrated superior agreement (mean: 97.3%), with diet beverages achieving exceptional concordance (99.3% within LoA) and plant-based milks showing minimal bias (0.9 mL, 98.6% within LoA, *p* = 0.316). Coffee and tea categories exhibited the largest absolute biases, particularly black coffee/tea (100.1 mL) and sweetened coffee/tea (68.2 mL), though still maintaining acceptable agreement (95.7% within LoA). These findings likely reflect the challenge of estimating frequently consumed, variable-portion beverages throughout the day. Alcoholic beverages (wine, beer, hard liquor) showed high agreement rates (94.3–99.3%), though interpretation should consider the low consumption frequency in this Turkish population, potentially influenced by cultural and religious factors. Critically, total SSB intake—the primary focus given the public health implications outlined in our introduction—demonstrated acceptable agreement for both volume (−243.4 mL bias, 96.5% within LoA) and energy (−31.4 kcal, 99.3% within LoA). Total beverage intake showed larger positive bias (641.1 mL) but maintained acceptable agreement (95.0% for volume, 98.6% for energy). The predominance of proportional bias across categories suggests that while BEVQ-15 provides acceptable agreement at population level, individual-level estimates may be less precise at extreme intake levels.

**Table 4:** Bland–Altman analysis results for agreement between the BEVQ-15 and 3-day dietary records across beverage categories.

Category	Mean bias	SD	Lower LoA	Upper LoA	% Within LoA	Proportional bias *p*
Water (mL)	−34.603	213.853	−453.755	384.55	97.2	<0.001
100% Fruit Juice (mL)	6.099	16.744	−26.719	38.918	94.3	0.0019
100% Fruit Juice (kcal)	3.319	9.852	−15.991	22.628	93.6	0.0026
Sweetened Fruit Drink (mL)	10.45	28.228	−44.878	65.777	94.3	<0.001
Sweetened Fruit Drink (kcal)	2.843	14.229	−25.047	30.732	94.3	0.0236
Whole Milk (mL)	9.051	33.531	−56.67	74.771	97.9	<0.001
Whole Milk (kcal)	5.863	21.635	−36.54	48.267	96.5	<0.001
Low-Fat Milk (mL)	13.39	49.857	−84.331	111.111	97.2	<0.001
Low-Fat Milk (kcal)	4.25	17.045	−29.159	37.658	95.7	<0.001
Nut Milk (mL)	0.908	24.833	−47.766	49.581	98.6	0.316
Nut Milk (kcal)	0.249	7.999	−15.429	15.927	98.6	0.4306
Regular Soft Drink (mL)	13.704	47.879	−80.138	107.547	93.6	0.0121
Regular Soft Drink (kcal)	2.63	30.018	−56.205	61.465	93.6	0.0572
Energy & Sports Drinks (mL)	1.794	8.17	−14.22	17.807	93.6	0.2734
Energy & Sports Drinks (kcal)	0.852	3.821	−6.638	8.341	93.6	0.2523
Diet Sweetened Drinks (mL)	10.946	94.161	−173.61	195.501	99.3	0.0001
Diet Sweetened Drinks (kcal)	0.109	0.942	−1.736	1.955	99.3	0.0001
Sweet Tea (mL)	2.855	17.815	−32.063	37.772	97.2	<0.001
Sweet Tea (kcal)	6.266	43.923	−79.823	92.355	97.2	<0.001
Coffee/Tea Black (mL)	100.052	284.746	−458.051	658.154	95.7	0.0006
Coffee/Tea Black (kcal)	−0.251	1.187	−2.578	2.076	90.1	<0.001
Coffee/Tea + Milk/Cream (mL)	68.228	119.253	−165.508	301.965	95.7	<0.001
Coffee/Tea + Milk/Cream (kcal)	16.203	28.494	−39.646	72.052	94.3	<0.001
Wine (mL)	2.049	17.904	−33.043	37.141	96.5	<0.001
Wine (kcal)	−0.127	17.749	−34.914	34.661	95.7	0.7209
Hard Liquor (mL)	6.715	76.208	−142.652	156.082	99.3	<0.001
Hard Liquor (kcal)	18.582	171.823	−318.192	355.356	99.3	<0.001
Beer (mL)	2.853	44.848	−85.048	90.755	94.3	0.0543
Beer (kcal)	−0.854	14.62	−29.51	27.801	94.3	0.6473
Total SSB (mL)	−243.355	544.677	−1310.922	824.212	96.5	<0.001
Total SSB (kcal)	−31.445	220.659	−463.937	401.048	99.3	<0.001
Total Beverage Intake (mL)	641.143	588.239	−511.805	1794.092	95	<0.001
Total Beverage Intake (kcal)	98.732	291.531	−472.669	670.134	98.6	<0.001

## 4 Discussion

Beverage consumption can contribute significantly to an individual’s total daily energy intake, particularly through high-calorie beverages, which are closely associated with the development of obesity. Therefore, accurately assessing beverage intake is essential for estimating beverage-derived energy intake. This need highlights the importance of employing valid and reliable tools to measure beverage consumption in individual and population-level dietary assessments (17). The present study aimed to evaluate the validity and reliability of the BEVQ-15 following its cultural and linguistic adaptation into Turkish. The findings indicated that the scale demonstrates moderate validity and high test–retest reliability in assessing habitual beverage intake.

Correlation analyses between the BEVQ-15 and 3-day dietary intake records revealed consistent and strong associations across all time points for the categories of water, nut milk, energy and sports drinks, diet or artificially flavored sweetened drinks, and sweet tea. These findings indicate that the BEVQ-15 can accurately and reliably reflect habitual consumption patterns in these beverage categories. When compared with the BEVQ-15 update study conducted with adults over 18 years of age (16), the high correlations observed in both studies for water and diet or artificially flavored sweetened drinks suggested that the questionnaire provides consistent and valid results across different cultural populations for these beverage types. In contrast, while our study identified strong correlations for nut milk and energy and sports drinks, the original BEVQ-15 study (16) reported low correlations for these categories. This discrepancy may be attributed to increased awareness and familiarity with nut milk and energy and sports drinks in Türkiye, which may have enabled participants to record and report their consumption more accurately. Moderate correlations were observed for 100% fruit juice, sweetened fruit drink, whole milk, reduced fat milk or chocolate milk, low-fat or fat-free milk, buttermilk or soy milk, regular soft drinks, coffee/tea black, wine, and total beverage intake. Collectively, these findings suggest that the Turkish version of the BEVQ-15 effectively captures habitual consumption across a wide range of beverage types. The predominance of high and moderate correlations indicates that the questionnaire is comprehensible for participants and largely reflects their actual beverage consumption patterns.

The findings related to the test–retest reliability of the BEVQ-15 demonstrated excellent reliability for repeated measures of water intake and moderate reliability for diet or artificially sweetened beverages. This result indicates that participants reported their intake of these beverages consistently over time, suggesting that the habitual consumption patterns of these categories are well captured by the questionnaire. Moderate levels of reliability were observed for 100% fruit juice, sweetened fruit drink, whole milk, reduced fat milk or chocolate milk, low-fat or fat-free milk, buttermilk or soy milk, regular soft drinks, energy and sports drinks, coffee/tea black, beer, total SSBs, and total beverage intake, which implies that although some variation exists across time points, the BEVQ-15 provides reasonably consistent estimates of intake for these beverage types. In contrast, low test–retest reliability was found for nut milk, sweet tea, coffee/tea sweetened/milk/cream, wine, and hard liquor. This finding may be attributable to the infrequent or irregular consumption of these beverages, participants’ difficulty in estimating portion sizes, or challenges in recording consumption over a given period. In the 15-item update study, consistency was measured by administering the BEVQ at two different times and assessing reproducibility. All 15 beverage categories, total SSB, and total beverage intake were found to be significantly correlated and consistent across both measures (16). In a validation study of the BEVQ with Spanish adults over the age of 18, ICC values between the BEVQ-15 and the food frequency questionnaire were found to be low to moderate. Furthermore, high reliability was demonstrated, as evidenced by a Cronbach’s *α* of 0.981 (18). Taken together, the results suggest that while the BEVQ-15 demonstrates high reliability for frequently and regularly consumed beverages, its reliability is more limited for beverage categories with less defined consumption patterns. This finding highlights the potential influence of consumption frequency and record accuracy on the reliability of self-reported beverage intake data.

Correlation and regression analyses are frequently used to evaluate the agreement between two quantitative measurements. However, correlation is not recommended for comparisons because it presents the relationship between two methods and does not evaluate the differences. Bland–Altman analysis can be used as an alternative to evaluate the agreement between two quantitative measurements by examining the mean difference and establishing the limits of agreement (19). Considering the literature, we performed Bland–Altman analysis in addition to correlation analysis. We found that the BEVQ-15 showed good agreement in predicting beverage intake compared with the 3-day food record, with most differences falling within acceptable limits. We also determined that total SSB intake showed acceptable compliance for both volume and energy. However, the spread of data points increased at higher mean intake levels, suggesting greater variability for higher beverage consumption. This proportional bias pattern, commonly observed in dietary assessments, may reflect underreporting in record methods or overestimation in BEVQ-15 responses for large quantities. This finding demonstrates the difficulty in estimating variable portion sizes of beverages consumed frequently throughout the day. The 15-item update study showed slightly below-acceptable agreement between the BEVQ-15 and dietary records for total beverage intake (94 and 96%, respectively) and SSB intake (94 and 92%, respectively) (16). In the Spanish validation study, agreement was assessed with kappa instead of Bland–Altman, and kappa values ranged from 0.823 to 0.929. All scores were very close to 1, indicating a high level of agreement (18). In general, Bland–Altman plots showing ≥95% consensus indicate acceptable agreement between instruments (19, 20). Beverage intake questionnaires are likely to yield slight differences compared to dietary records. In sum, considering the findings of both our study and the original scale (15), the BEVQ has been demonstrated to be a repeatable and valid tool that is comparable to dietary records for estimating beverage consumption.

This study has several limitations that warrant consideration. First, reliance on self-reported methods (BEVQ-15 and 3-day food records) may introduce recall and social desirability biases—particularly for SSBs—despite dietitian review. Second, the sample was predominantly female, highly educated, relatively young, and recruited from a single nutrition clinic in Istanbul, which may limit generalizability to other Turkish populations (e.g., older adults, lower education levels, clinical cohorts). Third, seasonality and reactivity cannot be excluded: data collection occurred in summer months (May–July), and the 15-day retest interval may have heightened intake awareness, potentially affecting subsequent reports. Fourth, low consumption frequency for several beverage categories (notably alcoholic drinks in this context) constrains precision in evaluating measurement properties and may reflect cultural and religious patterns rather than instrument deficits. Fifth, evidence of proportional bias in multiple categories suggests error may increase with intake volume, implying that the instrument may be more suitable for ranking individuals than for precise absolute intake estimation in epidemiologic applications. Finally, criterion validity against objective biomarkers (e.g., urinary sugars) and head-to-head comparisons with alternative Turkish beverage assessment tools were not conducted, and the absence of a definitive gold standard for habitual beverage intake remains an overarching challenge in nutritional epidemiology.

## 5 Conclusion

The Turkish version of the BEVQ-15 demonstrated satisfactory psychometric performance, providing evidence of both validity and reliability for assessing habitual beverage intake among adults. The instrument performed particularly well for water, diet beverages, and sugar-sweetened beverage assessment—key categories for public health surveillance. This validated questionnaire offers a standardized and culturally appropriate method to support future nutritional epidemiology studies, monitor beverage consumption trends, and inform targeted public health interventions. Overall, the findings confirm the utility and suitability of the BEVQ-15 for quantifying beverage intake in the Turkish adult population.

## Data Availability

The raw data supporting the conclusions of this article will be made available by the authors, without undue reservation.
